# Impact of COVID-19 on Obesity Management Services in the United Kingdom (The COMS-UK study)

**DOI:** 10.1007/s11695-020-05005-1

**Published:** 2020-10-13

**Authors:** Osama Moussa, Roxanna Zakeri, Chanpreet Arhi, Mary O’Kane, Vanessa Snowdon-Carr, Vinod Menon, Kamal Mahawar, Sanjay Purkayastha, On behalf of the PanSurg collaborative (COVESITY)

**Affiliations:** 1 Division of Surgery and Cancer, Imperial College London, London, W2 1NY UK; 2 Department of Surgery, University College London Hospital, London, UK; 3 Department of Surgery, University Hospital Lewisham, London, UK; 4 Department of dietetics, Leeds Teaching Hospitals NHS Trust, Leeds, UK; 5 Clinical Psychology, Somerset NHS Foundation Trust, Leeds, UK; 6 Department of Bariatric surgery, University Hospitals Coventry & Warwickshire NHS Trust, Coventry, UK; 7 Department of Bariatric Surgery, Royal Sunderland Hospital, Sunderland, UK

**Keywords:** Pandemic, Obesity, Bariatric surgery, COVID-19

## Abstract

Coronavirus Disease-2019 (COVID-19) has had a severe impact on all aspects of global healthcare delivery. This study aimed to investigate the nationwide impact of the pandemic on obesity management services in the UK in a questionnaire-based survey conducted of professionals involved in the delivery. A total of 168 clinicians took the survey; the majority of which maintained their usual clinical roles and were not redeployed except physicians and nurse specialists. Nearly all (97.8%) elective bariatric surgery was cancelled, 67.3% of units cancelled all multidisciplinary meeting activity, and the majority reduced clinics (69.6%). Most respondents anticipated that the services would recommence within 1–3 months. This study found that the COVID-19 pandemic has had a severe impact on the services involved in the management of patients suffering from severe, complex obesity in the UK

## Introduction

Coronavirus Disease-2019 (COVID-19) is a respiratory disease caused by the novel Severe Acute Respiratory Syndrome coronavirus 2 (SARS-CoV-2) that reached pandemic status. While COVID-19 affects all groups, the risks of severe pathology and mortality appear higher in the older population and those with underlying comorbidities.

Since the UK Health and Social Care Act reforms of 2012, obesity management has been restructured within the UK NHS; hence NHS England and the Department of Health have issued guidelines regarding the commissioning of care for severe and complex obesity. A 4-tier model of care is accepted [1] increasing stepwise from community weight management, to finally bariatric surgery, as appropriate. Both the specialist weight management (commonly known as tier 3 services) and bariatric surgery services (tier 4 services) have multidisciplinary teams (MDT) delivering specialised care to this group of patients. [1, 2]

The COVID-19 pandemic has had a challenging effect on all healthcare systems. It is essential to quantify the loss of elective services to be able to mitigate them. The purpose of this study was to evaluate the impact of the COVID-19 pandemic on obesity management services in the UK.

## Methods

A descriptive, cross-sectional survey design was used to explore the impact of the COVID-19 pandemic on tier 3 and tier 4 weight management services in the UK. A questionnaire-based survey (https://www.surveymonkey.co.uk/r/COMS%2D%2DUK) was conducted on healthcare professionals in these services. Healthcare professionals involved were surgeons, physicians, psychologists, psychiatrists, dieticians and specialist nurses. The survey link was freely shared on social media platforms and email/social media groups of surgeons, dieticians and also distributed through the PanSurg collaborative.

The questionnaire was divided into separate sections for individual clinicians, and all responses were reported as percentages of the separate clinician role and not of the total number of respondents.

## Results

**Fig. 1:**
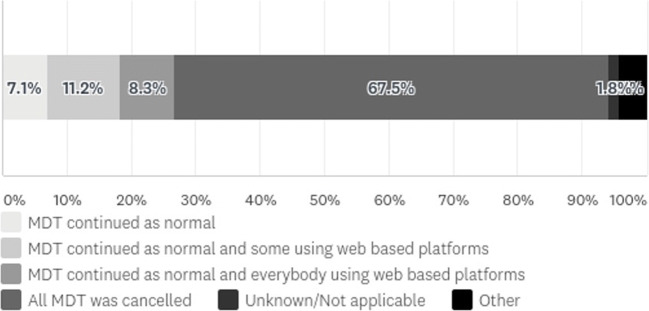
Impact of COVID-19 on MDT activity

A total of 168 clinicians took the survey. There were 53 surgeons (31.4%), 60 dieticians (35.5%), 20 psychologists/psychiatrists (11.8%), 14 physicians (8.3%) and 22 clinical nurse specialists (13.0%). The highest response was from London (18.5%); the lowest was from the North West and Oxford regions (1.8%). The majority (44.7%) worked in departments with an integrated tier 3 and 4 services. The highest response rate was from dieticians (35.5%) and bariatric surgeons (31.4%). Approximately 67.3% of units cancelled all (tiers 3 and 4) MDT meetings, but a minority (11.3%) continued remotely (Fig. 1).

### Surgeons

**Fig. 2:**
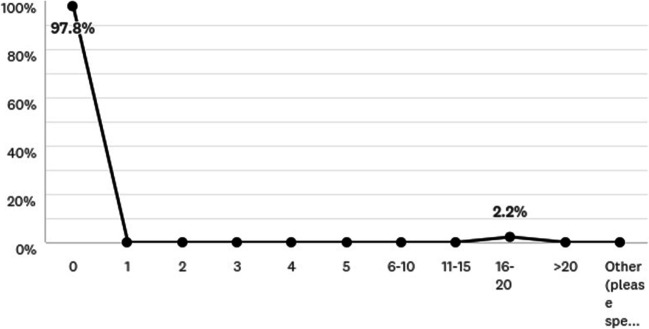
Impact of COVID-19 on elective bariatric procedures during April 2020

Bariatric surgical clinics were cancelled in 41.3% of tier 4 services, but telephone clinics continued in 15.9%. Most (97.8%) bariatric surgeons did not perform any procedures (Fig. 2) since the first 2 weeks of March (54.5%). The majority of bariatric surgeons (58.7%) were not reassigned but contributed to a general surgery role (80.4%), and continued a bariatric surgery emergency role (30.4%). 55.8% of surgeons performed a median of two emergency bariatric procedures during April 2020. COVID-19 did not impact decision-making for potential complications post-bariatric surgery in 61.4%, although 20.5% did, such as offering laparoscopy for suspected internal herniation (34.1%) or emergency gastric band removal (31.8%), however, would perform with full PPE.

### Dieticians

Half (50.9%) remained in their specialist weight management role. Most tier 4 clinics were reduced or cancelled (50%), while 78.8% were converted to telephone clinics. Dietetic advice was most necessary in postoperative patients (68.6%). Services relied on 86.5% of patients self-reporting their bodyweights. Postoperative haematological and biochemical investigations were generally not being monitored (48.1%). 59.6% relied on the general practitioner for nutritional supplementation prescribing, while 32.0% offered alternatives. 46.2% reported no additional dietetic support sought although 52% provided support through video and telephone contact. The majority (75.0%) were uncertain about relaxing preoperative weight loss targets, once surgery resumes.

### Psychology/Psychiatry

Overall, 40% of psychologists or psychiatrists continued to support patients, and only 25% were redeployed to a different role at the same site. Through the pandemic, 45% of psychologists reduced clinic capacity, of which 70% was changed to telephone clinics and 25% video clinics. Half (50%) felt that patients experienced a moderate adverse impact, detailed as preoperatively (55%), postoperatively (50%) and those with complex bariatric or revisional surgery (25%). The majority used online resources (60.0%), leaflets or brochures (30%), T.V. and video tutorials (25%) for mental health education.

### Physicians

Half (50%) were redeployed to different roles, while 25% continued their routine obesity services. Three quarters (75%) physicians cancelled all obesity specialist clinics, although telephone clinics (50%) and virtual/letter correspondence (25%) continued. Despite this, 50% continued remote monitoring of quantifiers such as blood glucose levels, HbA1C and blood pressure remotely.

### Nurse Specialists

Nurse specialists (42.1%) were redeployed but still occasionally undertaking bariatric surgery roles. Clinics were reduced or cancelled (40%), and 75% were converted to telephone clinics. This had a severe adverse impact on patients. Body weights were self-reported in 65% and 80% of reported no increase in patients seeking support.

## Discussion

The outbreak of COVID-19 (caused by SARS-COV-2) has had a detrimental effect on global healthcare systems. The World Health Organization (WHO) declared the COVID-19 outbreak as a global emergency on 30th January 2020 [3]. Balancing the risk for patients with mitigating health conditions and healthcare workers need to continue to provide effective treatment. Given that obesity alone is a significant cause of physical and mental health impairment, the additive effect of COVID-19 can also impact patients’ ability to sustain healthy lifestyle changes and routinely engage with their health care teams.

Overall, the UK bariatric services have primarily been reduced or cancelled across all disciplines throughout, as foreseen. Furthermore, we highlight the main structural changes that have emerged during the pandemic and possible long term alternatives. There are obvious limitations to this study mainly as a weakness of the strategy that the authors are unable to determine the actual response rate due to the use of social media for distribution; however, there were a large number of responses, and it is more than likely to have captured a representative sample.

Multidisciplinary meetings were predominantly cancelled during April. Hardly any elective surgery was carried out, but emergency services continued to be provided but reduced numbers. The change in services nationally was also highlighted in the recent prioritisation report [4]; however, the actual impact on services in the UK has not been gauged up to date.

Broadly, healthcare professionals in tier 3 and tier 4 services were mostly retained in their current roles and not redeployed except conceivably physicians and nurse specialists (Table 1). However, there was a significant impact on clinics (Table 2) that were mostly converted to remote telephone clinics (Table 3), across all roles. There were solutions offered remotely that have the potential to be considered in future services such as remote MDT meetings, weight monitoring, advice and supplementation as well as other means of dietetic and psychological assessment.

**Table 1:** Obesity management clinic impact during the month of April 2020 pandemic (percent ratio of individual speciality)

Obesity management clinics	All cancelled (%)	Some cancelled (%)	Continued as normal (%)	Unknown/not applicable (%)
Surgeons	41.3	37.0	15.2	6.5
Dieticians	17.31	50.0	9.6	23.0
Psychologists/psychiatrists	30.0	45.0	15.0	10.0
Physician	75.0	0.0	8.3	16.7
Nurse specialist	28.6	38.1	0.0	33.3

**Table 2:** Obesity management clinic adaptation during the month of April 2020 pandemic (percent ratio of individual speciality)

	Normal clinic (%)	Normal clinic (fewer patients) (%)	Video clinics (%)	Telephone clinics (%)	Virtual/Letter (%)	Not applicable (%)
Surgeons	2.2	2.2	8.7	69.6	6.5	10.9
Dieticians	0	0	3.9	78.9	3.9	13.4
Psychologists/psychiatrists	0	0	30.0	70.0	10.0	25.0
Physician	0	0	0	50.0	25.0	25.0
Nurse specialist	0	0	0	76.2	4.8	19.1

**Table 3:** Impact of COVID-19 on obesity management roles (percent ratio of individual speciality)

	Different role, same site (%)	Different role, different site (%)	Same obesity manage role (%)	Same role and different speciality (%)	Not applicable/other (%)
Surgeon	15.2	10.9	6.5	58.7	8.7
Dietician	23.1	1.9	51.9	5.8	17.3
Psychologists/psychiatrists	25.0	0.0	40.0	5.0	30.0
Physician	50.0	8.3	25.0	8.3	8.3
Nurse specialist	28.6	4.8	23.8	0	42.8

From the author’s experience alternate interim strategies that were considered also included strict remote dietician weight monitoring, continued psychologist support, scrutinising of biochemical markers through general practitioners or metabolic physicians and surgeons individually virtually communicating with high-risk patients. Also, pharmacological means were considered (such as Liraglutide), but sparse evidence supported this. Lastly, the UK government have recently revised obesity strategies on 27th July 2020 summarising the impact of alternate strategies in light of evidence gathered during the pandemic on the association between obesity, COVID-19 and mortality [5].

There was agreement amongst clinicians that obesity is a risk factor for contracting COVID-19 as well as associated with a higher risk of morbidity and mortality amongst, which has already been presented [6]. There is also a suggested increase in the risk of COVID-19 severe complications in patients with obesity, or obesity-associated comorbidities or both [6–8]. There has been limited evidence base that obesity has a significant direct association with acquiring the virus, furthermore, was not adjusted to BMI categories. Perioperative mortality has been described, however, not specific to bariatric procedures [9, 10].

When evaluating the insight into recommencing services, almost all clinicians advocated 1–3 months, similarly demonstrating a willingness to restart services before the pandemic completely subsides. This has also been highlighted with regard to which patient group should clinicians commence services with initially, as described recently by Rubino et al. [4]. It was felt that the slightly higher risk metabolic group should perhaps be addressed upon starting services with the recommended preoperative measures [11]. Within this study, there was a consensus to modify appropriate preoperative screening (83.7%) for COVID-19 as well as operating with full PPE (69.8%) (FFP3 or N95 mask), primarily ensuring appropriate COVID-19 swab screening (84.4%) and operating on a COVID-19 clean site (40.0%). There was additional interest in two negative nasopharyngeal swabs for viral PCR (43.5%), antibody test when available (54.4%), self-isolating for 2 weeks (63.0%) or a computed tomography scan (C.T.) chest (47.8%). There have been measures taken to restart bariatric surgery during the pandemic, British Obesity and Metabolic Surgical Society (BOMSS) guidance [12] on a safe recommencement and some units have already recommenced services within the UK. There are also recommendations from the Federation of Surgical Specialist Associations (FSSA) guidance [13].

This survey is a comprehensive general overview of one of the most rapidly growing burdens related to specialities. It is crucial to understand current practices at a time of controversy due to lack of best evidence-based practice. This brief overview has established the availability and willingness of such a crucial speciality to be restored.

## Conclusion

To the authors’ knowledge, this is the first published study that has grasped the current workforce distribution for obesity management services during the COVID-19 pandemic within the UK. The workforce and most units have been overall preserved, displaying the ability to manage the burden of the ongoing obesity epidemic remotely. There has been an ambition to recommence services despite the apparent risks, aiming to restrict the growth of another crucial disease pandemic. In this primary healthcare and economic crisis, ongoing obesity management remains vital to the health of present and future generations to come.
